# Treatment of Acne

**Published:** 1892-07

**Authors:** 


Treatment of Acne.—Dr. Wm. F. Waugh, in the *Times and Register*, summarizes his views on the treatment of acne as follows:

Correct any derangement of the general health which a thorough examination may disclose.

Regulate the general and personal hygiene.

The vigorous application of soft soap is indicated ie all cases except the rare acute form; but especially for infiltration.

Nearly all authors recommend sulphur ointments for ordinary cases; mercurials for severe ones.

Except when clearly indicated, internal remedies are rarely of use.

The disease is singularly obstinate; hence, changes in the treatment adopted after mature reflection should not be made except for cause.

Ergot will cause the indurations to disappear quickly; but they will return when the drug is discontinued.

The prolonged use of strychnine and nitro-muriatic acid sometimes effects a cure.

℞ Strychninæ sulphat................gr. j.
Acidi nitro-muriatici..............f ʒ iij.
Aquæ dest. q. s....................ad f ℥ v.

M. Sig.—A teaspoonful in water before each meal.

I have more than once failed to benefit a patient until he abstained from malt liquors.—*Gaillard's Med. Journal.*

